# Bacterial coinfection and antimicrobial use among patients with COVID-19 infection in a referral center in the Philippines: A retrospective cohort study

**DOI:** 10.1016/j.ijregi.2022.07.003

**Published:** 2022-07-08

**Authors:** Cybele L. Abad, Joanne Carmela M. Sandejas, Jonnel B. Poblete, Anna Flor G. Malundo, Maria Sonia S. Salamat, Marissa M. Alejandria

**Affiliations:** a Division of Infectious Diseases, Department of Medicine, University of the Philippines Philippine General Hospital, UP-Manila, Philippines; b Department of Medicine, University of the Philippines Philippine General Hospital, UP-Manila, Philippines

**Keywords:** Bacterial coinfection, COVID-19, antibiotic prescription

## Abstract

**Objective:**

This study aimed to describe community-acquired bacterial coinfection (CAI) and antimicrobial use among COVID-19 patients.

**Methods:**

Electronic records were retrospectively reviewed, and clinical data, laboratory data, antibiotic use, and outcomes of patients with and without CAI were compared.

**Results:**

Of 1116 patients, 55.1% received antibiotics within 48 hours, but only 66 (5.9%) had documented CAI, mainly respiratory (40/66, 60.6%). Patients with CAI were more likely to present with myalgia (*p* = 0.02), nausea/vomiting (*p* = 0.014), altered sensorium (*p* = 0.007), have a qSOFA ≥ 2 (*p* = 0.016), or require vasopressor support (*p* < 0.0001). Patients with CAI also had higher median WBC count (10 vs 7.6 cells/mm^3^), and higher levels of procalcitonin (0.55 vs 0.13, *p* = 0.0003) and ferritin (872 vs 550, *p* = 0.028). Blood cultures were drawn for almost half of the patients (519, 46.5%) but were positive in only a few cases (30/519, 5.8%). Prescribing frequency was highest at the start and declined only slightly over time. The mortality of those with CAI (48.5%) was higher compared with those without CAI (14.3%).

**Conclusion:**

Overall CAI rate was low (5.9%) and antimicrobial use disproportionately high (55.0%), varying little over time. The mortality rate of coinfected patients was high. Certain parameters can be used to better identify those with CAI and those who need blood cultures.

## Highlights

• The rate of COVID-19 and community-acquired coinfections was low.
• Those who are coinfected have higher mortality, and need to be identified early.
• Antibiotic use was disproportionately high and varied little over time.
• Blood cultures are low yield, and should not be performed routinely.

## Introduction

Coronavirus disease 2019 (COVID-19), an infection caused by severe acute respiratory syndrome coronavirus 2 (SARS-CoV-2), has resulted in a global pandemic infecting more than 271 million people worldwide. As of February 9, 2022, the cumulative number of cases and deaths in the Philippines had reached 3 623 176 and 54 690 respectively, based on the latest Department of Health COVID-19 dashboard.

Despite the viral origin of COVID-19, antibiotic therapy is often routinely given and blood cultures frequently drawn upon admission. Published data on the epidemiology of COVID-19 in the Philippines are scant (Abad et al., 2021; Edrada et al., 2020; Salamat et al., 2021; Soria et al., 2021) and no studies have looked at coinfection or prescription practices. Our study aimed to: 1) describe the profiles of COVID-19 patients with a community-acquired bacterial respiratory coinfection (CAI) or bacteremia; and 2) illustrate changes in antimicrobial use over time in a tertiary COVID-19 referral hospital.

## Methodology

### Study design and setting

A retrospective review of adult patients (> 19 years of age) with COVID-19 infection confirmed by reverse transcriptase-polymerase chain reaction (RT-PCR), and admitted to the University of the PhilippinesPhilippine General Hospital (UP-PGH), Manila, Philippines was conducted. The UP-PGH was designated by the Philippine Department of Health (DOH) as a COVID-19 hospital on March 30, 2020, with 26 ICU and 250 non-ICU beds dedicated to COVID-19 patients. The study was conducted in accordance with ethical guidelines and approved by the Institutional Review Board of the UP Manila (UPMREB CODE 2020-285-01).

### Data collection and study sample

Two study authors (JCS, JBP) retrospectively reviewed both written and electronic records — i.e. Registry of Admissions and Discharges (RADISH) and PGH Medical Record System (OpenMRS) — of consecutive COVID-19-confirmed admissions over a 6-month period (from March 12, 2020 to August 31, 2020), using a standardized data collection form. All data gathered were stored on a Microsoft Excel worksheet. Missing data, inconsistencies, and accuracy of information were reviewed.

**Fig. 1:**
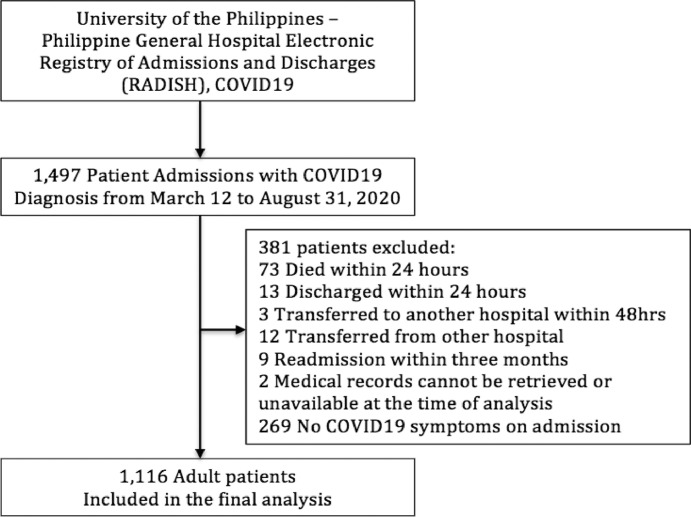
Study flow chart showing the selection process.

Patients who were asymptomatic, died, were discharged within 24 hours of admission, transferred to another hospital within 48 hours, transferred from another hospital, readmitted within 3 months of the patient's first admission, or whose medical records were not available for review during the time of analysis were excluded (Fig. 1).

### Study variables and definitions

Study variables included age, sex, comorbid illnesses, symptoms on presentation, baseline vital signs, diagnostic tests, and radiographic imaging. Clinical severity of COVID-19 on admission, receipt of antibiotics, and detailed microbiological data were also recorded.

*Confirmed COVID-19* was defined as any patient with a positive RT-PCR test for COVID-19. Based on existing guidelines, severity of COVID-19 illness was classified as follows: *mild —* presence of COVID-19 but without evidence of pneumonia; *moderate —* presence of COVID-19 symptoms and comorbidities such as hypertension, cardiovascular disease, diabetes mellitus, chronic obstructive pulmonary disease (COPD), asthma, or an immunocompromising condition (e.g. human immunodeficiency virus (HIV) infection, chronic steroid use, and active malignancy), or with pneumonia but without the need for oxygen support; *severe —* the presence of pneumonia, oxygen saturation < 92% on room air and requiring oxygen support; *critical* — COVID-19 infection with findings of acute respiratory distress syndrome (ARDS), septic shock, or the need for mechanical ventilation and/or ICU admission (PSMID, 2021; World Health Organization, 2020).

Coinfections were considered community acquired (CAI) if they were identified within the first 48 hours of hospitalization and confirmed via a positive culture. A bloodstream infection was considered a *true bacteremia* if a patient had a positive blood culture *and* clinical manifestations of infection (Horan and Gaynes, 2004). Sputum cultures were considered only if the sputum sample was of adequate quality (e.g. > 25 polymorphonuclear cells/low-power field (lpf) and epithelial cells < 10/lpf) (García-Vázquez et al., 2004; Geckler et al., 1977; van der Eerden et al., 2005) with growth of pathogenic bacteria (Shen and Sergi, 2022). A *contaminant* was defined as a microorganism not considered pathogenic to the patient. The following were considered contaminants if they were found only once in a set of blood cultures (e.g. 1 of 2 or 1 of 3 sets): coagulase-negative staphylococci (CoNS), *Propionibacterium acnes, Corynebacterium spp.* (diphtheroids), *Bacillus spp.*, α-hemolytic viridans group streptococci, and *Micrococcus spp.* (Dargère et al., 2018). A *colonizer* was defined as an organism found in or on the body but not causing any symptoms or disease — for example, *Candida* spp. isolated from respiratory or urine cultures. The authors evaluated all patients with positive cultures and reached consensus to determine clinical relevance, based on a review of the records. *Empiric antibacterial therapy* was defined as any antibacterial started within 48 hours of hospitalization, pending microbiological data. Antibiotics prescribed ≥ 48 hours from admission were considered treatment for hospital-acquired infection (Metlay et al., 2019) and were excluded.

### Blood and sputum collection methods

*Blood culture —* 5–10 milliliters (ml) of blood were drawn from two separate venipuncture sites or from a central venous catheter if indicated, and inoculated directly into two aerobic blood culture bottles up to the fill line. *Sputum culture —* sputum was either expectorated by the patient or induced with the assistance of a respiratory therapist, and collected using a sterile cup. All samples were processed following the Clinical and Laboratory Standards Institute M100 30th edition supplement (Clinical and Laboratory Standards Institute, 2020).

### Statistical analysis

Using descriptive statistics, frequency distributions of demographic and clinical characteristics for quantitative variables were determined. Median was used as the measure of central tendency in this patient population, with the interquartile range (IQR) of the quantitative variables provided for measures of dispersion. All tests were two-tailed, with *p*-value less than 0.05 considered statistically significant. Analysis was conducted using Microsoft Excel and MedCalc Statistical Software version 19.7.4.

## Results

### Demographics and clinical characteristics of the cohort

In total, 1116 were included patients in the study cohort. Around half were male (586, 52.5%) and the overall median age was 55 years (range 23–95). The majority of patients had one comorbidity (*n* = 803, 72%), with hypertension (HTN) being most common. Cough (696, 62.4%), shortness of breath (505, 45.2%), and fever (644, 57.7%) were the most common presenting symptoms. Close to half of patients presented with moderate COVID-19 (453, 40.6%), followed by critical (299, 26.8%), mild (192, 17.2%), and severe (172, 15.4%) (Table 1).

**Table 1:** Demographic and clinical profiles of patients with COVID-19 and those with community-acquired coinfection

	Overall	Community-acquired coinfection		*p*-value
	(*N* = 1116)	With (*N* = 66)	Without (*N* = 1050)	
**AGE**				0.158
Median (IQR)	55(23–95)	57.5 (45–66)	54 (47–67)	
< 60 years, No. (%)	687 (61.6)	35 (53.0)	652 (62.1)	
≥ 60 years, No. (%)	429 (38.4)	31 (47.0)	398 (37.9)	0.142
**SEX,** Male, No. (%)	586 (52.5)	36 (54.5)	550 (52.4)	0.732
**COEXISTING CONDITION,** No. (%)				
Presence of any comorbid illness	803 (72)	51 (77.3)	752 (71.6)	0.321
Hypertension	535 (47.9)	41 (62.1)	494 (47.0)	0.017
Diabetes mellitus	281 (25.2)	21 (31.8)	260 (24.8)	0.200
Heart disease	157 (14.1)	10 (15.2)	147(14.0)	0.794
Chronic kidney disease	97 (8.7)	9 (13.6)	88 (8.4)	0.142
Asthma	79 (7.1)	3 (4.5)	76 (7.2)	0.408
Neurological disease	78 (7)	6 (9.1)	72 (6.9)	0.490
Cancer	67 (6)	3 (4.5)	64 (6.1)	0.607
Active pulmonary tuberculosis	36 (3.2)	4 (6.1)	32 (3.0)	0.180
Chronic obstructive pulmonary disease	27 (2.4)	1 (1.5)	26 (2.5)	0.622
Chronic liver disease	9 (0.8)	0	9 (0.9)	0.450
Human immunodeficiency virus	7 (0.6)	0	7 (0.7)	0.506
**Symptoms,** No. (%)				
Cough	696 (62.4)	46 (69.7)	650 (61.9)	0.205
Fever	644 (57.7)	36 (54.5)	608 (57.9)	0.592
Shortness of breath	504 (45.2)	42 (63.6)	462 (44.0)	0.002
Malaise/fatigue	316 (28.3)	19 (28.8)	297 (28.3)	0.930
Diarrhea	187 (16.8)	11 (16.7)	176 (16.8)	0.983
Sore throat	176 (15.8)	10 (15.2)	166 (15.8)	0.887
Decreased appetite	148 (13.3)	16 (24.2)	132 (12.6)	0.007
Headache	88 (7.9)	2 (3.0)	86 (8.2)	0.131
Myalgia	87 (7.8)	7 (10.6)	80 (7.6)	0.380
Change or loss in taste	85 (7.6)	5 (7.6)	80 (7.6)	0.990
Decreased sensorium	81 (7.3)	12 (18.2)	69 (6.6)	0.0004
Change or loss in smell	79 (7.1)	3 (4.5)	76 (7.2)	0.408
Nausea or vomiting	54 (4.8)	9 (13.6)	45 (4.3)	0.0006
Chills	49 (4.4)	4 (6.1)	45 (4.3)	0.495
**Imaging, chest X-ray**, No. (%)	1110 (99.5)			
With pneumonia	752 (67.4)	54 (81.8)	698 (66.5)	0.010
Pulmonary infiltrates				
Bilateral	621 (55.6)	45 (68.2)	576 (54.9)	0.035
More than 50% of the lungs	428 (38.4)	38 (57.6)	390 (37.1)	0.001
Ground glass	541 (48.5)	38 (57.6)	503 (47.9)	0.001
Consolidation	96 (8.6)	7 (10.6)	89 (8.5)	0.550
Pleural effusion	88 (7.9)	7 (10.6)	81 (7.7)	0.397
**Severity of Illness**, No. (%)				
Mild	192 (17.2)	5 (7.6)	187 (17.8)	
Moderate	453 (40.6)	13 (19.7)	440 (41.9)	
Severe	172 (15.4)	13 (19.7)	159 (15.1)	
Critical	299 (26.8)	35 (53.0)	264 (25.1)	< 0.00001
qSOFA ≥ 2	92 (8.2)	15 (22.7)	68 (6.5)	0.0160
**Diagnostics,** median (IQR)				
*Complete blood count*				
Hemoglobin, g/L	132 (116–144)	127 (106–142)	132 (110–141)	0.0309
Hematocrit	40 (35–43)	38 (32–43)	40 (33–43)	0.0813
White blood cells, × 10^9^/L	7.7 (5.7–10.5)	10 (7.4–14.8)	7.6 (6–11.9)	< 0.0001
Neutrophils, %	69 (58–81)	84 (71–88)	69 (65–85)	< 0.0001
Lymphocytes, %	19 (10–29)	10 (5–19)	20 (8–23)	< 0.0001
Absolute lymphocyte count, cells/mm^3^	1363 (896–1937)	970 (660–1558)	1386 (750–1654)	0.0003
Platelets, × 10^9^/L	271 (202–354)	252 (185–328)	273 (186–356)	0.1306
*Arterial blood gas*				
pH	7.42 (7.39–7.46)	7.38 (7.29–7.43) *n* = 65	7.42 (7.39–7.46)	< 0.0001
pCO_2_	35 (29–39)	33 (28–40)	35 (27–37)	0.3454
pAO_2_	90 (76–106)	91 (78–137)	90 (70–110)	0.1167
HCO_3_	23 (19–25)	19 (16–22)	23 (17–24)	< 0.0001
PaO_2_ and FiO_2_ ratio	376 (252–456)	358 (187–451)	378 (175–419)	0.2724
*Chemistry*				
Blood urea nitrogen, mmol/L	5 (3.6–8.6)	7 (4–25)	5 (4–12)	0.0001
Creatinine, mmol/L	75 (56–113)	97 (65–329)	74 (59–141)	0.001
Estimated glomerular filtration rate, ml/min/1.73 m^2^	91 (56–109)	74 (13–97)	92 (41–103)	0.0005
Aspartate aminotransferase, IU/L	47 (32–75)	49 (29–73)	47 (36–88)	0.7487
Alanine aminotransferase, IU/L	38 (21–70)	35 (18–63)	38 (21–73)	0.2655
Albumin, g/dL	0.7 (0.5–0.9)	34 (30–39)	38 (31–40)	0.0051
Total bilirubin, mg/dL	0.3 (0.2–0.4)	0.7 (0.5–1)	0.7 (0.5–1)	0.3645
Direct bilirubin, mg/dl	0.4 (0.2–0.6)	0.4 (0.3–0.7)	0.3 (0.2–0.5)	0.0003
Indirect bilirubin, mg/dl	5.0 (3.6–8.6)	0.3 (0.09–0.5)	0.4 (0.2–0.6)	0.037
*Inflammatory markers*				
Lactate dehydrogenase, U/L	313 (237–475)	413 (284–625)	310 (285–581)	0.001
Ferritin, ng/ml	559 (202–1320)	872 (309–1630)	550 (361–1820)	0.0284
Procalcitonin, ng/mL	0.16 (0.04–0.56)	0.55 (0.06–4.03)	0.13 (0.08–0.8)	0.0003
D-dimer, µg/mL	1.33 (0.58–3)	3.6 (1.1–9)	1.3 (0.9–3.6)	< 0.0001
**Outcomes**				
Length of stay in days, median (IQR)	13 (8-20)	12 (6-19)	13 (8-20)	0.2767
Mortality, No. (%)	183 (16.4)	32 (48.5)	150 (14.3)	0.0001

Only 66 patients (5.9%) had a documented concomitant bacterial CAI — mainly respiratory (*n* = 40, 66.7%). Among those with CAI, the median age was 57.5 (range 45–66) years. Those with CAI were more likely to present with myalgias (7 vs 24, *p* = 0.024), nausea or vomiting (9 vs 32, *p* = 0.0136), and altered sensorium (13 vs 51, *p* = 0.007), compared to those without. Patients who had a concomitant CAI were likely to be more ill, with a qSOFA > 2 (*p* = 0.016), and require vasopressor support (*p* = 0.001), than those without a coinfection (Table 1).

Of those with a coinfection, around half were bacteremic (30/66, 45.4%). Bacteremic patients were more likely to have underlying hypertension (HTN) (*p* = 0.022) or chronic kidney disease (CKD) (p = 0.033), and to present with chills (*p* = 0.025), myalgia (*p* = 0.006), nausea or vomiting (*p* < 0.001), and tachypnea (*p* = 0.011). Median WBC count (11.3 vs 9 cells/mm^3^, *p* = 0.012) and procalcitonin level (2.96 vs 0.34 ng/ml, *p* < 0.001) were higher for those who were bacteremic (Table 2).

**Table 2:** Characteristics of patients with and without bacteremia

			BACTEREMIA				
	Overall		Positive		Negative		*p*-value
	1116		30		489		
	Median/*N*	IQR/%	Median/*N*	IQR/%	Median/*N*	IQR/%	

**AGE**							
Median, IQR	55		61.5	50–68	59	48–68	0.7381
Less than 60 years, No. (%)	687	61.6%	13	43.3%	253	51.7%	
60 years and above, No. (%)	429	38.4%	17	56.7%	236	48.3%	0.3718
**SEX,** No. (%)							
Male	586	52.5%	21	70.0%	294	60.1%	
Female	530	47.5%	9	30.0%	195	39.9%	0.2828
**COEXISTING CONDITION**, No. (%)							
Presence of any comorbid illness	803	72.0%	26	86.7%	393	80.4%	0.3963
Diabetes mellitus	281	25.2%	13	43.3%	134	27.4%	0.0604
Hypertension	535	47.9%	22	73.3%	254	51.9%	0.0228
Heart disease	157	14.1%	6	20.0%	86	17.6%	0.7372
Chronic liver disease	9	0.8%	0	0.0%	5	1.0%	0.5782
Chronic kidney disease	97	8.7%	8	26.7%	63	12.9%	0.0331
COPD	27	2.4%	1	3.3%	15	3.1%	0.9349
Asthma	79	7.1%	0	0.0%	25	5.1%	0.2047
Active pulmonary tuberculosis	36	3.2%	2	6.7%	23	4.7%	0.6263
HIV	7	0.6%	0	0.0%	3	0.6%	0.6673
Cancer	67	6.0%	2	6.7%	42	8.6%	0.714
Neurological disease	78	7.0%	5	16.7%	51	10.4%	0.2856
**SYMPTOMS**							
Headache	88	7.9%	0	0.0%	27	5.5%	0.1866
Chills	49	4.4%	4	13.3%	21	4.3%	0.025
Fever	644	57.7%	14	46.7%	306	62.6%	0.0822
Cough	696	62.4%	22	73.3%	336	68.7%	0.5956
Rhinorrhea/congestion	153	13.7%	3	10.0%	39	8.0%	0.6934
Shortness of breath	504	45.2%	21	70.0%	304	62.2%	0.3899
Sore throat	176	15.8%	2	6.7%	44	9.0%	0.6631
Myalgia	87	7.8%	4	13.3%	16	3.3%	0.0055
Malaise/fatigue/generalized weakness	316	28.3%	11	36.7%	146	29.9%	0.431
Diarrhea	187	16.8%	6	20.0%	74	15.1%	0.474
Nausea or vomiting	54	4.8%	8	26.7%	31	6.3%	<0.0001
Decreased appetite	148	13.3%	8	26.7%	94	19.2%	0.3198
Abdominal pain/discomfort	56	5.0%	2	6.7%	33	6.7%	0.9862
Change or loss in taste	85	7.6%	3	10.0%	27	5.5%	0.3081
Change or loss in smell	79	7.1%	1	3.3%	13	2.7%	0.8249
Decreased sensorium	81	7.3%	6	20.0%	55	11.2%	0.1489
**LABORATORY TESTS**							
Complete blood count, median (IQR)							
Hemoglobin	132.0	116 – 144	117	90–132	126	107– 140	0.0843
Hematocrit	40.0	35–43	35.5	28–42	38	33–43	0.1349
White blood cells	7.7	5.7–10.52	11.25	9.5–16.6	9	6.275–12.625	0.0121
Neutrophils	69.0	58–81	85	77–90.	78	69–86	0.0014
Lymphocytes	19.0	10–29	6.5	3–12	12	7–20	0.0006
Absolute lymphocyte count	1363.0	896.25–1937.75	805.5	498–1188	1050	698–1533.5	0.0257
Neutrophil lymphocyte ratio	3.6	2.070–7.9	13.19	7–29	6.25	3.49–12.29	0.0006
Platelets	271.0	202–354	210	160–295	256	181–356	0.0788
Blood chemistry, median (IQR)							
BUN (mmol/L)	5.0	3.6–8.6	19.1	6.725–40.8	6.35	4.3–14.2	0.0002
Serum creatinine (µmol/L)	75.0	56–113	192.5	97–788	85	61–158.25	0.0004
eGFR	91.0	56–109	34	5.000–73.000	78	33.75–101	0.0012
AST (U/L)	47.0	32–75	56	33.000–95.500	58	39– 93.5	0.7731
ALT (IU/L)	38.0	21–70	33	17.5–83	40	21–75	0.778
Total bilirubin ((mg/dl)	0.67	0.5–0.988	0.91	0.543–1.230	0.77	0.53–1.128	0.405
Direct bilirubin (mg/dl)	0.29	0.2–0.44	0.53	0.375–0.750	0.37	0.26–0.6	0.0026
Indirect bilirubin (mg/dl)	0.38	0.220–0.6	0.29	0.00250–0.555	0.365	0.2–0.63	0.054
Inflammatory markers, median (IQR)							
LDH (U/L)	313.0	237.5–475	474	322.000–744.500	428	307.25–635	0.3872
Serum ferritin (ng/mL)	559.0	202.75–1320	1135	646.500–1735.000	986.5	456–2050	0.6782
Serum procalcitonin (ng/mL)	0.16	0.04–0.56	2.96	0.548–13.260	0.34	0.123–1.11	< 0.0001
D-dimer (ug/mL)	1.33	0.58–3.012	3.7	1.670–7.320	1.955	1.095–3.875	0.0497
C-reactive protein, No. (%)							
No CRP determination	152	13.6%	1	3.3%	57	11.7%	
≤ 12 mg/L	374	33.5%	5	16.7%	67	13.7%	
> 12 mg/L	590	52.9%	24	80.0%	365	74.6%	0.3607
Mild	192	17.2%	0	0.0%	19	3.9%	
Moderate	453	40.6%	4	13.3%	127	26.0%	
Severe	172	15.4%	7	23.3%	116	23.7%	
Critical	299	26.8%	19	63.3%	227	46.4%	< 0.0001
**STATUS ON ADMISSION,** No (%)							
Requiring oxygen support	469	42.0%	25	83.3%	342	69.9%	
On ventilatory support	85	7.6%	12	40.0%	69	14.1%	
Acute respiratory distress syndrome	221	19.8%	10	33.3%	166	33.9%	0.9451
On vasopressor	27	2.4%	5	16.7%	19	3.9%	0.0012
qSOFA ≥ 2	92	8.2%	10	33.3%	72	14.7%	0.0067

### Diagnostics

#### Basic chemistry/serological tests

A complete blood count (CBC) was performed in the majority of patients (1081, 96.9%). Median white blood cell (WBC) and absolute lymphocyte counts (ALC) were 10 vs 7.6 cells/mm^3^ and 1386 vs 970 × 10^9^ cells/liter, for those with and without coinfection, respectively. Procalcitonin levels were measured in only about half of the patients (586, 52.5%); the median value was higher for those who had a CAI compared with those who did not (0.55 vs 0.13, *p* = 0.0003). Those with procalcitonin values accounted for 76/192 (39.6%), 232/453 (51.2%), 92/172 (53.4%), and 186/299 (62.2%) of those with mild, moderate, severe, and critical COVID-19 illness, respectively. Ferritin and lactate dehydrogenase (LDH) levels were higher among those with CAI compared to those without, at 872 vs 550 (*p* = 0.0284) and 413 vs 310 (*p* = 0.001), respectively.

#### Cultures

Cultures were ordered at the discretion of the healthcare team. Blood cultures were performed in about half of patients (519, 46.5%). These patients accounted for 19/192 (9.9%), 131/453 (28.9%), 123/172 (71.5%), and 246/299 (82.3%) of those with mild, moderate, severe, and critical COVID-19 illness, respectively. Only one-third were able to provide a sputum sample within 48 hours (331, 29%). These accounted for 26/192 (13.5%), 107/453 (23.6%), 65/172 (37.8%), and 133/299 (44.5%) of those with mild, moderate, severe, and critical COVID-19 illness, respectively. 135 bacterial and fungal species were isolated from 98 (8.8%) patients. In some instances, multiple organisms were isolated from blood (5/59), respiratory (10/65), or urinary (1/11) sites. Nearly half (44.1%, 26/59) of blood isolates were considered contaminants, while almost a quarter (23.1%, 15/65) of respiratory isolates were colonizers. The most common pathogen isolated from blood and treated as an infection was CoNS (*n* = 32). A breakdown of specific pathogens is provided in Supplementary Table 1.

### Antibiotic use and prescribing pattern

**Fig. 2:**
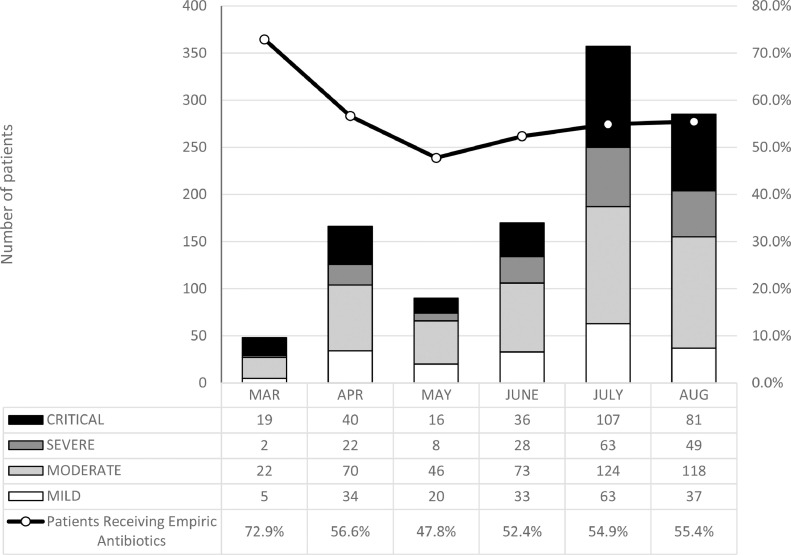
Monthly distribution of COVID-19 cases based on illness severity, compared with the monthly percentages of patients receiving empiric antibiotic treatment

More than half (614, 55.0%) of the cohort received empiric antibiotics on admission. The frequency of antibiotic prescribing by month was as follows: March (72.9%), April (56.6%), May (47.8%), June (52.4%), July (54.9%), and August (55.4%) (Fig. 2). Prescribing frequency increased according to severity of illness: 15.1%, 36.6%, 83.1%, and 92.3% for mild, moderate, severe, and critical disease, respectively (Supplementary Table 2).

Azithromycin (360, 35.1%), ceftriaxone (283, 27.6%), and piperacillin-tazobactam (250, 20.7%) were the most commonly prescribed antibiotics, with the majority (276, 92.3%) prescribed for patients with critical COVID-19. Antibiotics were given either as monotherapy (213, 19.1%) or, more often, as combination therapy (401, 35.9%) (Supplementary Table 2).

### Outcomes

All patients with mild COVID-19 recovered and were discharged. Length of hospital stay was similar between those who were coinfected and those who were not. Overall mortality for those with coinfections was higher compared with those without coinfections — 32/66 (48.5%) vs 150/1050 (14.3%), *p* < 0.0001 (Table 1).

## Discussion

In our cohort, the overall rate of documented CAI was low at 5.9%, and antimicrobial use disproportionately high at 55%, which was consistent with other reports (Garcia-Vidal et al., 2021; Lansbury et al., 2020; Musuuza et al., 2021; Vaughn et al., 2021). However, our study highlights several other findings: first, patients with a concomitant bacterial infection were more likely to present with myalgia, altered sensorium, higher WBC, and higher procalcitonin levels; second, trends in antimicrobial use did not vary over time despite changes in recommendations (Langford et al., 2021); third, routine blood cultures were low yield; and finally, mortality rate was higher among those who were coinfected compared with those who were not.

Empiric antibiotics are often prescribed among patients with COVID-19 because of the possibility of coinfection. In theory, empiric therapy covers for bacterial community-acquired pneumonia (CAP), and testing both sputum and blood is considered when disease is severe or there is concern for multidrug-resistant (MDR) pathogens (Metlay et al., 2019; Wu et al., 2020). Despite low rates of documented bacterial CAI, our study showed that over half (55.0%) of hospitalized patients received empiric antibiotics upon admission. This was slightly lower but comparable with pooled data from across the globe, which showed rates of empiric antibiotic use ranging from 72% to almost 100% (Cao J. et al., 2020; Chen et al., 2020; Huang et al., 2020; Wang D. et al., 2020). Ironically, antimicrobial misuse drives antimicrobial resistance (Roca et al., 2015), and following antibiotic stewardship principles even in the context of a pandemic is crucial to avoid the emergence of resistance (Majumder et al., 2020).

Initial guidelines for COVID-19 management recommended early use of antibiotics in all suspected COVID-19 cases with sepsis. This was evident in our study, with the highest rate of antimicrobial prescription (79%) during the beginning of the epidemic in March. The uncertainty of treating a novel illness also likely contributed to this high rate of antibiotic use. As understanding about COVID-19 evolved, however, routine antimicrobial use was discouraged (Langford et al., 2021). In our cohort, the lowest prescribing rate was in May (47.8%), although it remains uncertain as to which factors contributed to the slight improvement in antimicrobial prescribing practices over time. Not surprisingly, those who presented with more severe disease were given anti-infectives more frequently, with antibiotic use in over 90% of patients with severe or critical COVID-19 disease. Although it is difficult to withhold antimicrobials from those who are acutely ill, stewardship principles can still be followed — discontinuation of antimicrobials when both procalcitonin and WBC are normal, or when cultures are negative, should be considered. Alternatively, de-escalation to targeted treatment should be pursued. Whether these principles were followed should be addressed by future studies.

In this study, macrolides were the most frequently prescribed empiric antibiotic, in contrast with other studies, in which fluoroquinolones were more frequently prescribed (Cao B. et al., 2020; Langford et al., 2021; Wang D. et al., 2020; Wang Z. et al., 2020). Azithromycin, believed to have both antiviral activity and an immunomodulatory effect against COVID-19 (Echeverría-Esnal et al., 2021), was used frequently in March (24/48, 50.0%) but had gradually declined by August (87/285, 30.5%). Its benefits for COVID-19 were disproven around that time (RECOVERY Collaborative Group, 2021), which likely explains the decline in its use.

Blood cultures were taken in almost half the cohort (46.5%), but were positive in only a few cases (30/519, 5.7%). The most frequent organism isolated from blood was CoNS, which may not have always indicated a true coinfection. In one study (Hughes et al., 2020), a high proportion of blood culture contamination was due to unfamiliarity with personal protective equipment worn by healthcare workers. Thus, the low yield of blood cultures found in our study suggest that these should not be performed routinely, and the growth of Gram-positive cocci should be interpreted with caution. This is extremely relevant in a low–middle-income country such as the Philippines, where financial resources and health insurance coverage may be limited. Antimicrobials should also be withheld unless the clinical picture is compatible with bacteremia. In our study, patients with HTN or CKD, or those with chills, myalgia, nausea/vomiting, or tachypnea, were more likely to be bacteremic. Elevated WBC count and procalcitonin levels were also predictive of bacteremia, in line with another study (He et al., 2021). Procalcitonin levels may also help identify COVID-19 patients with bacterial coinfection (Williams et al., 2021) when used in combination with clinical assessment and other inflammatory markers (Peters et al., 2021).

The overall mortality rate among those in our cohort with bacterial CAI was higher than in those without coinfection (48.5 vs 14.3%). This validates a recent meta-analysis, which showed that patients with a coinfection or superinfection had higher odds of dying than those who only had SARS-CoV-2 infection (odds ratio = 3.31, 95% CI 1.82–5.99) (Musuuza et al., 2021). Interestingly, patients with moderate-to-critical COVID-19 who received empiric antibiotics had a higher mortality rate than those who did not (Supplementary Fig. 1). Although it is more likely that this was related to disease severity and prolonged hospitalization (Giske et al., 2008; Sydnor and Perl, 2011) rather than antibiotic use per se, this warrants further analysis.

Our study had several limitations inherent to its retrospective nature: relevant information on prior cultures, antimicrobial use, or initial empiric antimicrobial therapy may not have been captured accurately. Tests such as sputum or blood cultures, and procalcitonin, were left to the discretion of the healthcare team, and may have led to ascertainment bias (e.g. those with more severe illness were more likely to undergo testing). Moreover, our study was only able to document culture-based coinfections, underestimating the true incidence of CAI, because PCR-based tests (e.g. respiratory panels) are not routinely performed in our setting. Nevertheless, despite these limitations, our study involved a large sample size and was the first to focus on bacterial CAI and the pattern of antimicrobial use during the first 6 months of the pandemic in the country.

In summary, our study confirmed that antimicrobial use was high and varied little over time, despite a low rate of documented bacterial CAI among patients with COVID-19. The mortality rate of those who were coinfected was high, and so early identification is paramount. Specific clinical and diagnostic parameters can help determine the presence of a bacterial CAI, and thus guide decisions on performing blood cultures or beginning empiric antibiotic therapy.

## Ethical approval statement

This study was carried out in accordance with The Code of Ethics of the World Medical Association (Declaration of Helsinki) for experiments involving humans. Informed consent was waived, and the study was approved by the Institutional Review Board of UP-PGH.

## Research transparency and reproducibility

Data sets are available as supplementary material and from the authors upon reasonable request.

## Declaration of Competing Interest

All authors report no conflicts of interest relevant to this article.

## Appendix Supplementary materials

Image, application 1

Image, application 2
