# Supplementary material for: Bacterial coinfection and antimicrobial use among patients with COVID-19 infection in a referral center in the Philippines: A retrospective cohort study
Source: IJID Reg. 2022 Jul 8;4:123–30. doi: 10.1016/j.ijregi.2022.07.003 (PMC9263707; doi:10.1016/j.ijregi.2022.07.003)
Supplement: Supplementary file 1 [file mmc1.docx]

**Supplementary Figure:** Mortality of patients with and without empiric antibiotic therapy, according to severity of illness
