# Supplementary material for: Bacterial coinfection and antimicrobial use among patients with COVID-19 infection in a referral center in the Philippines: A retrospective cohort study
Source: IJID Reg. 2022 Jul 8;4:123–30. doi: 10.1016/j.ijregi.2022.07.003 (PMC9263707; doi:10.1016/j.ijregi.2022.07.003)
Supplement: Supplementary file 2 [file mmc2.docx]

Supplementary Table 1. Specific Pathogens According to Site of Isolation

|  | Blood | | | Respiratory | | | Other Sites (Urine, Ij Catheter Tip, Stool, Endocervical, Rectovaginal Swab) | | | Overall | | |
| --- | --- | --- | --- | --- | --- | --- | --- | --- | --- | --- | --- | --- |
| **Description** | Pathogen | Contaminant | Drug Resistant | Pathogen | Colonizer | Drug Resistant | Pathogen | Colonizer | Drug Resistant | Pathogen | Contaminant/Colonizer | Drug Resistant |
| **Gram-positive organisms** |  |  |  |  |  |  |  |  |  |  |  |  |
| *Streptococcus agalactiae* | 1 | 0 | 0 | 0 | 0 | 0 | 0 | 0 | 0 | 1 | 0 | 0 |
| *Streptococcus pneumoniae* | 0 | 0 | 0 | 1 | 0 | 0 | 0 | 0 | 0 | 1 | 0 | 0 |
| *Streptococcus viridans* | 1 | 0 | 0 | 0 | 0 | 0 | 0 | 0 | 0 | 1 | 0 | 0 |
| *Aerococcus viridans* | 1 | 0 | 0 | 0 | 0 | 0 | 0 | 0 | 0 | 1 | 0 | 0 |
| *Staphylococcus aureus* | 2 | 0 | 0 | 2 | 0 | 1 | 0 | 0 | 0 | 4 | 0 | 1 |
| *Staphylococcus epidermidis* | 8 | 3 | 3 | 0 | 0 | 0 | 0 | 0 | 0 | 8 | 3 | 3 |
| *Staphylococcus haemolyticus* | 5 | 2 | 2 | 0 | 0 | 0 | 0 | 0 | 0 | 5 | 2 | 2 |
| *Staphylococcus hominis* | 18 | 11 | 9 | 0 | 0 | 0 | 0 | 0 | 0 | 18 | 11 | 9 |
| *Staphylococcus urealyticus* | 1 | 0 | 0 | 0 | 0 | 0 | 0 | 0 | 0 | 1 | 0 | 0 |
| *Enterococcus faecium* | 0 | 0 | 0 | 0 | 0 | 0 | 1 | 0 | 1 | 1 | 0 | 1 |
| Diphtheroids | 6 | 6 | 0 | 0 | 0 | 0 | 1 | 1 | 0 | 7 | 7 | 0 |
| *Bacillus spp* | 4 | 4 | 0 | 0 | 0 | 0 | 0 | 0 | 0 | 4 | 4 | 0 |
| **Sub total** | 47 | 26 | 14 | 3 | 0 | 1 | 2 | 1 | 1 | 52 | 27 | 16 |
| **Gram-negative organisms** |  |  |  |  |  |  |  |  |  |  |  |  |
| *Citrobacter koseri* | 0 | 0 | 0 | 1 | 0 | 1 | 0 | 0 | 0 | 1 | 0 | 1 |
| *Enterobacter aerogenes* | 1 | 0 | 0 | 0 | 0 | 0 | 0 | 0 | 0 | 1 | 0 | 0 |
| *Enterobacter cloacae* | 0 | 0 | 0 | 1 | 0 | 0 | 0 | 0 | 0 | 1 | 0 | 0 |
| *Enterobacter hormaechei* | 0 | 0 | 0 | 2 | 0 | 0 | 0 | 0 | 0 | 2 | 0 | 0 |
| *Escherichia coli* | 1 | 0 | 1 | 0 | 0 | 0 | 4 | 0 | 3 | 5 | 0 | 4 |
| *Klebsiella pneumoniae* | 3 | 0 | 0 | 26 | 0 | 5 | 0 | 0 | 0 | 29 | 0 | 5 |
| *Raoultella ornithinolytica* | 0 | 0 | 0 | 1 | 0 | 0 | 0 | 0 | 0 | 1 | 0 | 0 |
| *Proteus mirabilis* | 1 | 0 | 0 | 0 | 0 | 0 | 0 | 0 | 0 | 1 | 0 | 0 |
| *Serratia marcescens* | 1 | 0 | 0 | 0 | 0 | 0 | 0 | 0 | 0 | 1 | 0 | 0 |
| *Acinetobacter baumannii* | 2 | 0 | 0 | 8 | 0 | 6 | 0 | 0 | 0 | 10 | 0 | 6 |
| *Acinetobacter lwoffii* | 1 | 0 | 0 | 0 | 0 | 0 | 0 | 0 | 0 | 1 | 0 | 0 |
| *Burkholderia cepacia* | 0 | 0 | 0 | 0 | 0 | 0 | 0 | 0 | 0 | 0 | 0 | 0 |
| *Comamonas testosteroni* | 0 | 0 | 0 | 1 | 0 | 1 | 0 | 0 | 0 | 1 | 0 | 1 |
| *Delftia acidovorans* | 1 | 0 | 0 | 0 | 0 | 0 | 0 | 0 | 0 | 1 | 0 | 0 |
| *Pseudomonas aeruginosa* | 0 | 0 | 0 | 7 | 0 | 1 | 0 | 0 | 0 | 7 | 0 | 1 |
| *Stenotrophomonas maltophilia* | 0 | 0 | 0 | 0 | 0 | 0 | 0 | 0 | 0 | 0 | 0 | 0 |
| **Sub total** | 11 | 0 | 1 | 47 | 0 | 14 | 4 | 0 | 3 | 62 | 0 | 18 |
| **Fungi** |  |  |  |  |  |  |  |  |  |  |  |  |
| *Candida albicans* | 0 | 0 | 0 | 6 | 6 | 0 | 1 | 1 | 0 | 7 | 7 | 0 |
| *Candida dublinensis* | 0 | 0 | 0 | 2 | 2 | 0 | 0 | 0 | 0 | 2 | 2 | 0 |
| *Candida famata* | 0 | 0 | 0 | 2 | 2 | 0 | 0 | 0 | 0 | 2 | 2 | 0 |
| *Candida glabrata* | 0 | 0 | 0 | 0 | 0 | 0 | 1 | 0 | 0 | 1 | 0 | 0 |
| *Candida tropicalis* | 1 | 0 | 0 | 5 | 5 | 0 | 3 | 1 | 0 | 9 | 6 | 0 |
| **Sub total** | **1** | **0** | **0** | **15** | **15** | **0** | **5** | **2** | **0** | **21** | **17** | **0** |
| **Total** | **59** | **26** | **15** | **65** | **15** | **15** | **11** | **3** | **4** | **135** | **44** | **34** |

Supplementary Table 2. Antimicrobial Use According to Severity of Illness

| **Antibiotic Class** | **Overall antibiotic prescribed,**  **N (%)** | **Mild, n=192** | **Moderate, n=453** | **Severe, n=172** | **Critical, n=299** |
| --- | --- | --- | --- | --- | --- |
| **MONOTHERAPY** |  |  |  |  |  |
| Beta lactam (Amoxicillin-clavulanate, Ampicillin-sulbactam, Piperacillin-Tazobactam) | 180 (84.5) | 10 | 55 | 32 | 83 |
| Macrolide (Azithromycin) | 12 (5.6) | 4 | 3 | 1 | 4 |
| Lincomycin (Clindamycin) | 1 (0.5) | 0 | 0 | 0 | 1 |
| Carbapenem (Ertapenem, Meropenem) | 20 (9.4) | 1 | 1 | 6 | 12 |
| Cephalosporins (Cefuroxime, Ceftriaxone, Cefoxitin, Cefixime, Ceftazidime, Cefepime) | 0 | 0 | 0 | 0 | 0 |
| Aminoglycoside (Amikacin) | 0 | 0 | 0 | 0 | 0 |
| Fluoroquinolone (Levofloxacin, Ciprofloxacin) | 0 | 0 | 0 | 0 | 0 |
| Nitroimidazole (Metronidazole) | 0 | 0 | 0 | 0 | 0 |
| Sulfonamides (Trimethoprim-Sulfamethoxazole) | 0 | 0 | 0 | 0 | 0 |
| Glycopepetide (Vancomycin) | 0 | 0 | 0 | 0 | 0 |
| Antifungal (Fluconazole) | 0 | 0 | 0 | 0 | 0 |
| **Subtotal** | 213 | 15 | 59 | 39 | 100 |
| **COMBINATION** |  |  |  |  |  |
| Beta lactam + Macrolide | 342 (85.3) | 12 | 93 | 93 | 143 |
| Beta lactam + Lincomycin | 12 (3.0) | 0 | 5 | 1 | 6 |
| Fluoroquinolone + Nitroimidazole | 1 (0.2) | 1 | 0 | 0 | 0 |
| Beta lactam + Macrolide + Lincomycin | 1 (0.2) | 0 | 1 | 0 | 0 |
| Beta lactam + Macrolide + Nitroimidazole | 3 (0.7) | 1 | 1 | 0 | 1 |
| Beta lactam + Fluoroquinolone | 20 (5.1) | 0 | 3 | 2 | 15 |
| Beta lactam + Nitroimidazole | 3 (0.8) | 0 | 2 | 1 | 0 |
| Carbapenem + Nitroimidazole + Antifungal | 1 (0.2) | 0 | 0 | 1 | 0 |
| Carbapenem + Fluoroquinolone | 5 (1.2) | 0 | 0 | 2 | 3 |
| Carbapenem + Glycopeptide | 3 (0.8) | 0 | 0 | 1 | 2 |
| Carbapenem + Glycopeptide + Nitroimidazole | 1 (0.2) | 0 | 0 | 1 | 0 |
| Beta lactam + Aminoglycoside | 3 (0.8) | 0 | 0 | 0 | 3 |
| Beta lactam + Fluoroquinolone + Lincomycin | 1 (0.2) | 0 | 0 | 0 | 1 |
| Beta lactam + Sulfonamide | 1 (0.2) | 0 | 1 | 0 | 0 |
| Beta lactam + Glycopeptide | 1 (0.2) | 0 | 0 | 1 | 0 |
| Beta lactam + Glycopeptide + Macrolide | 2 (0.6) | 0 | 0 | 1 | 1 |
| Beta lactam + Glycopeptide + Fluoroquinolone | 1 (0.2) | 0 | 0 | 0 | 1 |
| Subtotal | 401 | 14 | 107 | 104 | 176 |
| Overall | 614 (55.0%) | 29 (15.1%) | 166 (36.6%) | 143 (83.1%) | 276 (92.3%) |
